# Catalog of metagenome-assembled genomes of prokaryotic communities from the Red Sea hydrothermal vents

**DOI:** 10.1128/mra.00482-26

**Published:** 2026-08-04

**Authors:** Júnia Schultz, Sharifah Altalhi, Antônio Pedro Camargo, Nikos C. Kyrpides, Alexandre Soares Rosado

**Affiliations:** 1 Biological and Environmental Sciences and Engineering Division, King Abdullah University of Science and Technology 127355, Thuwal, Saudi Arabia; 2 Center for Nuclear Energy in Agriculture, University of São Paulo 67713, Piracicaba, Brazil; 3 Taif University 125895, Taif, Saudi Arabia; 4 DOE Joint Genome Institute, Lawrence Berkeley National Laboratory 1666, Berkeley, California, USA; Montana State University, Bozeman, Montana, USA

**Keywords:** metagenomics, microbial diversity, hydrothermal system, microbial genomes, extreme environment

## Abstract

This study presents medium- and high-quality prokaryotic metagenome-assembled genomes (MAGs) from microbial mats and sediments at Hatiba Mons, a Red Sea hydrothermal system. We recovered 1,217 bacterial and archaeal MAGs across 75 phyla, dominated by *Planctomycetota* and *Thermoproteota*. Approximately 70% of these genomes likely represent previously uncharacterized taxa.

## ANNOUNCEMENT

The exploration of hydrothermal vent ecosystems has revealed insights into the diversity of life in extreme environments (1, 2). However, Red Sea hydrothermal systems remain underrepresented in genome-resolved studies. The recent discovery of the first active hydrothermal vent fields in the Red Sea offers new opportunities to elucidate the composition and functional potential of microbial communities inhabiting these systems (3, 4). Here, we report 1,217 medium- and high-quality prokaryotic metagenome-assembled genomes (MAGs) recovered from Hatiba Mons, Red Sea rift.

A total of 31 samples (19 sediments and 12 microbial mats) were collected from 5 hydrothermal vents (22.28°N, 37.84°E) during expeditions in May and September 2022 and February 2023. Sediment samples were collected using gravity corers, while microbial mats were sampled using push cores, Niskin bottles, and box corers, in triplicate. All samples were immediately frozen at −20°C onboard and subsequently stored at −80°C in the laboratory until processing. Further details are available in Altalhi et al. (4). DNA was extracted from 10 g of sample using the DNeasy PowerMax Soil Kit (Qiagen, Germany), following the manufacturer’s protocol, with the addition of proteinase K (40 µL of 20 mg/mL, Omega) during lysis phase. DNA concentration was measured using the Qubit dsDNA HS Assay on a Qubit 4.0 fluorometer (Thermo Fisher Scientific, USA). For shotgun sequencing, extracted DNA ranging from 80 to 895 ng per sample was submitted to Novogene Inc. (California, USA) that prepared the library using the NEBNext Ultra II DNA Library Prep Kit (New England Biolabs, USA). Library preparation included DNA fragmentation, end repair, 5′ phosphorylation, A-tailing, adapter ligation, and PCR amplification. The resulting libraries were sequenced on an Illumina NovaSeq platform, generating paired-end 150-bp reads.

Metagenomic sequencing generated a total of 3,004,009,760 paired-end reads across all samples (ranging from 76,449,854 to 303,487,370 reads per sample). Low-quality reads and adapters were removed using fastp v1.3.0 (5). Quality-filtered reads were assessed with FastQC v0.12.1 and assembled using MEGAHIT v1.2.9 (6), resulting in 31 individual assemblies and 11 co-assemblies. Contigs ≥ 1,000 bp were binned using MetaBAT2 v1.7 (7), MaxBin2 v2.2.7 (8), and VAMB v5.0.2 (9), followed by bin aggregation with DAS Tool v1.1.7 (10). Genome quality was assessed using CheckM2 v1.1.0 (11), and only high-quality and medium-quality MAGs (12) were retained for downstream analyses. These bins underwent taxonomic assignment via GTDB-Tk v2.4.1 (release 226) (13). Protein-coding sequences were predicted from all MAGs using Prodigal (14). Default parameters were applied for all software.

**Fig 1:**
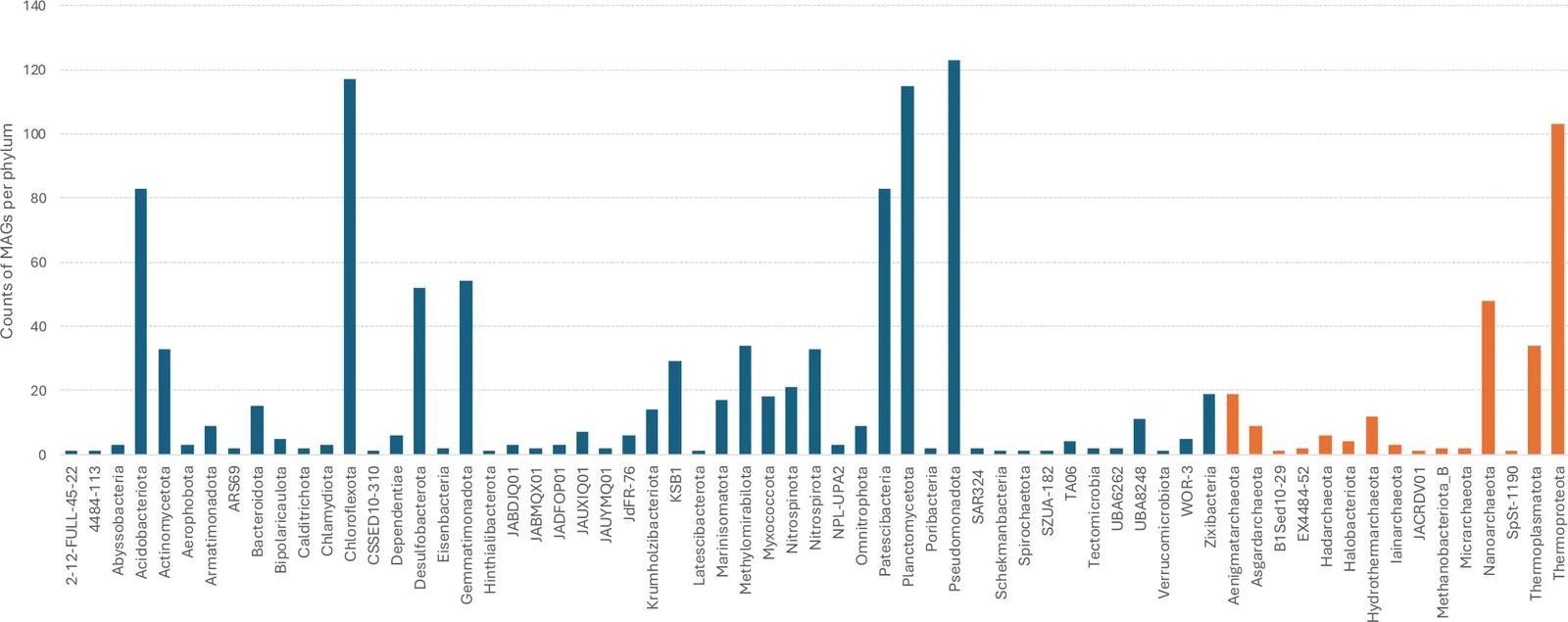
Distribution of metagenome-assembled genomes (MAGs) across prokaryotic phyla. The bar plot shows the number of recovered MAGs assigned to each bacterial (blue) and archaeal (orange) phylum based on GTDB taxonomy.

A total of 1,217 dereplicated prokaryotic MAGs were recovered from microbial mats and sediments. Of these, 969 bacterial MAGs spanned 50 phyla, with *Pseudomonadota*, *Chloroflexota*, and *Planctomycetota* as the most abundant groups. Additionally, 248 archaeal MAGs were identified, distributed across 15 phyla, with *Thermoproteota*, *Thermoplasmatota*, and *Nanoarchaeota* as the dominant taxa (Fig. 1). Based on GTDB RED (relative evolutionary divergence), approximately 70% of the MAGs likely represent novel taxa, a proportion that exceeds or matches reports from other hydrothermal vent systems. These findings highlight the Hatiba Mons as a hotspot of microbial novelty and expand available genomic references.

## Data Availability

The metagenomic data were deposited in the National Center for Biotechnology Information database under BioProject PRJNA1121395. BioSample accession numbers for the raw reads are from SRX24841039 to SRX24841069. MAG sequences and supplementary tables (metadata, quality, and taxonomy) are available in the open repository Zenodo (https://doi.org/10.5281/zenodo.19235833).
